# Viewpoint discrimination and contestation of ideas on its merits, leadership and organizational ethics: expanding the African bioethics agenda

**DOI:** 10.1186/1472-6939-14-S1-S1

**Published:** 2013-12-19

**Authors:** Sylvester C Chima, Takafira Mduluza, Julius Kipkemboi

**Affiliations:** 1 Programme of Bio & Research Ethics and Medical Law, Nelson R Mandela School of Medicine & School of Nursing and Public Health, College of Health Sciences, University of KwaZulu-Natal (UKZN), Durban, South Africa; 2 Department of Biochemistry, University of Zimbabwe, P.O. Box MP 167, Mount Pleasant, Harare, Zimbabwe; 3 Department of Biological Sciences and the UNESCO Regional Center for Documentation and Research on Bioethics, Egerton University, P.O Box 536-20115, Egerton, Kenya

**Keywords:** Africa, argumentation, bioethics, leadership, organizational ethics, structural violence, viewpoint discrimination, vulnerable populations

## Abstract

The 3^rd ^Pan-African Ethics Human Rights and Medical Law (3^rd ^EHRML) conference was held in Johannesburg on July 7, 2013, as part of the Africa Health Congress. The conference brought together bioethicists, researchers and scholars from South Africa, Zimbabwe, Kenya and Nigeria working in the field of bioethics as well as students and healthcare workers interested in learning about ethical issues confronting the African continent. The conference which ran with a theme of "Bioethical and legal perspectives in biomedical research and medical practice in Africa with a focus on: Informed consent, HIV-AIDS & Tuberculosis, leadership & organizational ethics, patients and healthcare workers rights," was designed to expand the dialogue on African bioethics beyond the traditional focus on research ethics and the ethical dilemmas surrounding the conduct of biomedical research in developing countries. This introductory article highlights some of areas of focus at the conference including issues of leadership, organizational ethics and patients and healthcare workers rights in Africa. We analyze the importance of free speech, public debate of issues, argumentation and the need to introduce the teaching and learning of ethics to students in Africa in accordance with UNESCO guidelines. This article also focuses on other challenges confronting Africa today from an ethical standpoint, including the issues of poor leadership and organizational ethics which are main contributors to the problems prevalent in African countries, such as poverty, poor education and healthcare delivery systems, terrorism, social inequities, infrastructural deficits and other forms of 'structural violence' confronting vulnerable African communities. We believe that each of the eight articles included in this supplement, which have been rigorously peer-reviewed are a good example of current research on bioethics in Africa, and explore some new directions towards broadening the African bioethics agenda as we move forward to a new dawn for Africa in the 21^st ^century.

## The importance of free speech and the contestation of ideas on merits in Africa

According to Jackson J in *CIO v Douds*, 'progress generally begins in skepticism about accepted truths' [1], yet when skepticism cannot be expressed, re-examination of established ideas and consideration of new ones will probably never occur. Under such circumstances intellectual contestation is fundamentally unfair and impairs the competition of ideas on its merits [2]. Concurring with this point of view, Scalia J has stated in *R.A.V v City of St. Paul *that when one side of a debate is allowed to fight freestyle, while the other is required to follow Marquis of Queensbury rules, under such circumstances, the contest is fundamentally unfair and impairs the competition of ideas on merits [3]. One of the problems plaguing Africa today seems to be the inability to freely express, analyze or accept different viewpoints without resorting to political intrigue or tribal and national sentiments as opposed to the merits or demerits of the different point of view. One of the reasons for this state of affairs may be due to the history of colonialism, neo-colonialism, ethnic complexities and multilingualism which were exploited as a divide and rule mechanism by previous colonial masters in Africa. The importance and power of listening to different points of view and free speech cannot be overemphasized and the recognition of this fact by western democracies has led to important constitutional amendments in developed countries such as the USA, where the first amendment to the US constitution was designed to protect the freedom of speech. The first amendment to the United States constitution is part of the citizen's Bill of Rights, and prohibits enactment of any law infringing on the freedom of speech, freedom of the press, interfering with the right of peaceful assembly by citizens, or prohibiting the petitioning for a governmental redress of grievances [4]. Therefore the first amendment while protecting the freedom of speech also provides the legal basis for barring laws based on viewpoint discrimination. In its ordinary application, neither the US Congress nor local authorities may pass laws that effectively "silence" one side in a political or social dispute [5]. Viewpoint discrimination occurs when a law specifically shows prejudice toward a particular point of view. For example, a regulation commits viewpoint discrimination where it only "attacks a particular individual's or group's message, as opposed to the mode in which that message is conveyed" [6]. Such laws are considered prima facie unconstitutional and are considered an egregious form of content discrimination based on the rights enshrined within the first amendment. Therefore, US Courts have consistently held such laws to be unconstitutional, while recognizing some exceptions to the provisions protecting freedom of speech. Speech that involves incitement, false statements of fact, obscenity, child pornography, offensive speech, threats, and speech owned by others are all completely exempt from first amendment protections, while commercial advertising receives diminished protection. These exceptions make the right to freedom of speech a limited one [7]. Because of these debates and controversies surrounding acceptable exceptions to free speech, viewpoint discrimination related cases have represented a significant area of United States constitutional law going back to the 18th century [6]. The practical impact of viewpoint discrimination, on the dissemination of information generally includes the problem that promotion of a particular viewpoint at the expense of others may create an active force of public persuasion creating a false and unbalanced impression [8]. It has been suggested that by picking one side or point of view and prohibiting expression of any other, those in power generally increase the chance that undecided citizens will adopt the preferred view and that it will influence public opinion and policy. For example, when one side of established thinking on any particular issue is favorably presented to the public and then hangs in the air, people might simply assume the proposition to be true or otherwise uncontestable because no critical or opposing views are allowed. Moreover, when a point of view bears the official imprimatur of those in power, some may take that fact in itself as proof of the idea's legitimacy [2]. The need to listen to different points of view and the importance of free speech and the debate of new ideas in Africa today is of the utmost importance if African countries are to take their place in the committee of nations in the 21st century.

## Argumentation and the importance of teaching and learning ethics

The ability to contest ideas in a rational and systematic manner is one of the benefits acquired through teaching and learning ethics. The study of ethics is important not only for individual lives, but also for developing the insight and competence African countries need, including a widespread educational curriculum on ethics, in order to face the challenges of the present and the future in a successful way. One of the skills students learn from ethics is the art of argumentation. Argumentation is a methodology for offering a set of reasons or evidence to support a particular conclusion or point of view. A good argument is supposed to provide evidence and give us good reasons to believe 'rational arguments' as opposed to a set of statements designed to sway an opponent e.g. in advertising. To distinguish between good and bad arguments and to be able to construct good rational arguments is something one must learn [9]. Rational arguments and rational dialogue are of the utmost importance for a well-functioning democracy. Argumentation is of crucial importance for fruitful discussions and learning, therefore it should be one of the main aims of the teaching of ethics to scientists, current students and future leaders in Africa, and should be included as part of a diverse course of programmes in colleges and universities in Africa. Emphasis on arguments is important not just out of consideration for the autonomy of the self but in recognizing and accepting the '*otherness of the others' *[10]. It is also an important part of social ethics. For example, *'emphasizing arguments will make life more difficult for political leaders and fanatics who spread messages which do not stand up to critical scrutiny, but which nevertheless often have the capacity to seduce the masses into intolerance and violence' *[9]. Three reasons have been outlined for the importance of argumentation in teaching and learning ethics: (a) Arguments are a way of finding out which views are better than others. (b) Arguments stimulate inquiry, in arguing for or against an issue, very often we discover that various factors are relevant to that issue, factors not thought about, but which may be crucial to explore. (c) Arguments also demonstrate respect for the otherness of others. Therefore we approach the other as an autonomous being, capable of making up his or her own mind, not as an entity to be manipulated by rhetorical devises, appeals to tribal or political authority, or other strategies [9].

## *Sumus ergo sum*- 'we are therefore I am'- the philosophy of Ubuntu

It has been argued that African ethics and cultural ethos are based on a different form of autonomy [11], such as communalism or Ubuntu which recognizes the needs and rights of the community above individual rights [12]. In the run up to the 3^rd ^EHRML, there was a call to boycott the conference by a segment of the medical fraternity in South Africa, based on the supposed need to defend the rights of one individual, as opposed to the needs of the community for the intellectual discourse and practical benefits of the medical conference and exhibition to the general public and local healthcare workers in particular [13]. Opposition to this point of view was met with vehement criticism [14], much of which was based on an incomplete understanding of ethical issues and the claiming of rights within the law [15]. While it has been argued that there is lack of policy debate in African bioethics because of the paucity of democratic policies and practices that promote freedom of thought, as well as lack of political transparency and accountability. Because of this it has been suggested that bioethics in Africa must become more political [16]. However, it needs to be recognized that the type of political activism being advocated by those who have made such calls is not the politics of individualism as supported by the advocates of boycotting the EHRML [14]. Rather, the type of political activism required by bioethicists in Africa is a political activism which promotes rights of the majority against the narrow individual self-interest and the rights of powerful minority groups [15-18]. The politics of individual privilege only serves to promote and perpetuate the structural inequities which are prevalent in Africa today [19]. Especially in countries like South Africa, which have a history of severe marginalization of the majority, while promoting the rights and interests of the minorities, in areas such as the distribution of healthcare services, wealth, education and access to scientific knowledge and all its accompanying benefits [17,18]. It has been suggested that such social inequities are what create the conditions for the persistence of poverty, disease, terrorism and other forms of structural violence [19], which reduces the chances of those who are less powerful from having access to improved quality of healthcare services and other social benefits [18-20]. A recent alternative world health report has suggested that failures of the current global economic system and its narrow focus on the disorders affecting the minority [21], rather than the rights and diseases facing the majority of the world population, otherwise known as the 10/90 anomaly, where 90% of global healthcare expenditure are spent on 10% of the worlds' population [8], has led to a situation where global health has not improved as it should, leading some authorities to argue for health as a right for everyone rather than a privilege for the wealthy few [21]. To put this in context of the EHRML, an advocacy to boycott a medical conference which is designed to improve the quality of healthcare for the majority should never be hijacked for the benefit of a few privileged individuals or political interest groups. Such attempts must be resisted by all fair minded persons who are concerned with promoting equality and reducing the social inequities which are prevalent in African communities and other vulnerable population groups [22,23].

## The problem of leadership in Africa

It has been argued that one of the major problems confronting Africa and contributing to its underdevelopment is the problem of leadership or lack thereof, leading the late author and Africanist, Chinua Achebe, to conclude in one of his books that "the trouble with Nigeria is simply and squarely a failure of leadership"[24], a statement which can be extended to include most African countries today. There are many styles of leadership as outlined in the keynote address by JM Mathooko at the 3rd EHRML conference [25]; it can be argued that one of the persistent problems of African leadership is that Africa has not been blessed by many servant leaders or a particularly visionary leadership in recent postcolonial history. The enviable attributes and advantage of servant leaders is that they focus on serving others, whether as employees, customers or the community at large, rather than self. Other qualities of servant leaders are that they have the capacity to listen and empathize. They are also persuasive and have a good awareness of their community or subjects needs. More importantly, servant leaders have the gift of foresight or vision with the capacity to conceptualize, and a commitment to stewardship and growth of their people and communities. Unfortunately, Africa seems be overwhelmed by the prevalence of leaders who see the opportunity to lead as an opportunity to promote individual self-interest and personal enrichment, at the expense of communal needs, thereby creating a situation whereby leaders in Africa have mostly brought on all forms of 'structural violence' on their communities [26-30], resulting in for example, failures within the healthcare delivery system, leading to such issues high levels of maternal and childhood mortality, rising incidence of infectious disease such as HIV/AIDS and tuberculosis [29,30], increased incidence of healthcare workers and public service strikes and poor education [31]. As a result African countries and their citizens' exhibit all major characteristics of vulnerable population groups as defined by UNAIDS and others [22].

## Organizational ethics -doing things the right way and the right way of doing things

One of the many problems of Africa can be associated with the lack of functional and effective organizational ethics. Organizational ethics became not only the ethical buzzword of the Western world in the late 20^th ^century, but began to infuse healthcare organizations (HCOs) after the call by the joint commission for accreditation of healthcare organizations (JCAHO) in the USA in the early 70s [32]. However, the use and application of organizational ethics is not limited to HCOs. The term covers a wide area of organizational culture, including the conduct of business affairs; decision-making processes; actual and potential conflicts of interest; appropriate use and allocation of financial, technological, and human resources; and the relation of the organization's actual activities to its stated mission and values [33]. It also deals with issues of organizational architecture which refers to ethical decision making, performance evaluation and compensation of employees [34]. Proponents of organizational ethics within HCOs contend that an organization's ethical responsibilities differ in kind from the responsibilities of individuals who work for it or who are associated with it. According to Paul Schyve the paradigm foundation of organizational ethics is not only each practitioner's obligation to the patient, a focus that has dominated healthcare ethics from the time of Hippocrates. More recently, a second source of obligation has arisen with the growing recognition that patients have "rights" in their relationships with healthcare providers. This dual recognition of professional obligations and patients' rights has fostered awareness in the commission's (JCAHO) statements and standards that organizations have an obligation to act to support and respect patients' rights [35]. On the other hand, simply locating organizational ethics in the personal ethics of each practitioner and administrator leaves too much to the vicissitudes of individual character. Based on this, Schyve concluded that HCOs have only one real choice: they must respond to new ethical challenges associated with managed care in the 21^st ^century [35]. While JCAHO has focused on the development of organizational mechanisms to address ethical problems in healthcare, Van Rensselaer Potter, the eminent scientist who coined the term 'bioethics' argues that, even in so-called organizational ethics, "individuals bear the responsibility" [36]. Potter is convinced that bioethics must also reach into all organizations, including public corporations and HCOs. However, he does not agree with the assignment of moral agency and responsibility to an organization itself, arguing that organizational "systems" and "processes," are the product of human action, and individuals cannot take moral refuge by laying blame on non-personal systems and processes [36]. This observation is very important for public organizations in Africa where the general attitude according to anecdotal evidence is that 'government business is nobody's business', an attitude which supports the kleptomania and corruption which are prevalent in public institutions in Africa today [27], generally characterized by laissez faire leadership [25] and personal gratification.

### Individual virtue and organizational ethics

The central focus of virtue ethics is the character of the person. In the context of medical ethics, virtue ethics is concerned with the characteristics of a good doctor, and in the context of organizations it is concerned with good leadership. According to Aristotle, "virtue is a state of character, concerned with choice lying in a mean, that is, the mean relative to us" [37]. Therefore, an action is considered right if, and only if, it is what a virtuous agent would do in the circumstances. Where a virtuous agent is one who exercises good virtues; and virtue is a character trait which human beings need in order to flourish. Therefore virtue refers to the character of individuals, characteristics such as honesty, integrity, respect, courage, truth-telling, persistence, nobility etc. In the context of 'organizational ethics', virtue would refer to character of the individuals within the organization, especially organizational leadership. According to some arguments every organization has goals and a 'way of doing things' that may appear 'ethical' or 'unethical'. Institutional goals therefore shape its core values. Nevertheless, Potter reminds us that an organization's goals and culture came from somewhere. Therefore an organization's leaders and key staff cannot avoid the burden of responsibility for the organization's actions and moral climate. They must be proactive by identifying potential ethical problems, addressing them before they arise and responding appropriately when the organization acts unethically [36].

### Improving organizational ethics in Africa

If organizations are to be guided by their missions while recognizing the need to improve profitability, then keeping a focus on the mission could direct leadership of such an organization to treat their employees with respect by acknowledging tough times and enlisting employee's aid in finding ways to achieve organizational goals. Therefore it has been suggested that organizations and individuals need each other to be ethical [33]. While medical ethics has been traditionally preoccupied with the interaction of doctors and their patients, vis-à-vis, the doctor-patient relationship, modern medical practice frequently occurs in a background of the non-medical management of HCOs, insurance and pharmaceutical companies who are driven by the profit motive [31]. This means that the ethical quality of health care may be broadly influenced by the ethics within HCOs, including healthcare insurance and pharmaceutical companies, other business entities or governments that are involved in the funding of healthcare, especially in the era of commercialization. In this context it has been postulated that "we can't have ethical health care without ethical organizations" [38]. It has been suggested that ethics within organizations can be addressed at several levels. These have been grouped into four categories as follows: (a) at the personal level, where issues affecting work-life balance, personal taxes etc. may need to be addressed. (b) At the organizational level where issues faced by employees and leadership such as strikes, retrenchment and cost cutting need to be addressed. (c) At the industrial level where situations encountered by professionals within organizations such as doctors, nurses, accountants, CEOs etc. need to be addressed. (d) Finally at the societal and external level where such as issues as globalization and climate change encountered by organizational leadership may need to be addressed [39]. All of these situations which can lead to conflicts in situational ethics create moral and ethical dilemmas within organizations associated with ethics as the "business of being human" [40]. Resolving these moral and ethical dilemmas within organizations especially in Africa, will require a systematic approach using well-established ethical guidelines such as utilitarianism, justice, rights, virtue ethics and principlism, rather than non systematic and non-rational approaches such as obedience, imitation, intuition, habit [41] or tribalism and nepotism. Factors which may lead to unethical behavior within organizations include the moral climate of society and the moral culture of business and industry [39]. Recent examples would include the unethical moral climate within the banking industry which led to the near collapse of the international banking enterprise in 2008 and the ENRON saga in the United States [42]. Questionable behaviors within such organizations which may have led to moral decline include amoral decision-making, unethical practices such as accepting illegality as standard behavior, lack of ethical leadership, overemphasis on profits and perceived pressure to meet goals, insensitivity towards subordinates and victimization of whistleblowers, together with inadequate formal ethics policies [39].

Therefore improving the ethical climate within organizations would include the introduction of written ethical codes and guidelines with distribution of these materials to all employees. These should be reinforced through frequent communications and training of employees and leadership, as well as the availability of ombudsmen and whistle-blower mechanisms for anonymous reporting of transgressions [39]. A general rule of thumb suggested for dealing with ethical dilemmas and moral obligations within organizations is that when two or more moral obligations are in conflict, it is advisable to use the stronger one. Further, when two or more ideals conflict or when ideals conflict with obligations, its best to honour the more important one. Finally, when there is an equal conflict between moral obligations or ideals, choose the one that produces the greatest good for the greatest number of people based on utilitarian principles [39]. To put in another way, one must always remember that though there may be many solutions to a particular ethical or moral dilemma. There are usually acceptable and non-acceptable solutions. Therefore, one must try always to choose the most acceptable solution when confronted with a particular moral or ethical dilemma in practice [41].

## The challenges of global ethics today and the way forward

According to Potter "bioethics means the application of ethics to all life" [43,44], however it has been suggested that in its early decades, ethics has been overlying focused on the interpersonal relationships between doctors/patients and researchers/human subjects of research, as well as new challenges related to the application of new biotechnologies to medicine and the health sciences [23]. This somewhat narrow focus has led bioethics to ignore larger societal concerns such as health inequalities within and between nations and the policies needed to reduce them and to promote fundamental globally social good [8]. This has led to a call that bioethics needs to broaden the agenda to other socio-political issues in society [23]. In Africa however, bioethics is still at its rudimentary stage due to excessive focus on the area of research ethics and the researcher/subject relationship [23,44,45]; especially in the context of western researchers coming to Africa and other developing countries to prospect for new drugs and conduct clinical trials [45]. Leading some African bioethicists to erroneously conclude that bioethics is flourishing in Africa simply because they have managed to produce a national policy for research ethics or once a few research ethics committees (RECs) have been established within some countries. While the emphasis on research ethics is important to protect human subjects of biomedical research, it is also equally important to expand the debate on African bioethics to include problems which are indigenous to Africa. Such problems associated with developing countries, include social inequities in economics and healthcare, justice, corruption, causes of climate change, poor education and infrastructure, poverty and other broadly defined elements of 'structural violence' [16,19]. All of which lead to social upheavals, such as frequent strikes and terrorism, etc. [26-31]. It has been suggested that the most important ethical dilemmas the world is facing today arise in connecting scientific research and the development and application of new technologies [9]. These new developments have consequences for almost all aspects of modern life including social media, globalization, cultural pluralism, development of new weapons, and terrorism including bioterrorism, climate change, depletion of natural resources and environmental deterioration including toxic waste dumping, deforestation and Genetically Modified Organisms (GMOs) [9]. It must be recognized that many of the possibilities opened up by new scientific breakthroughs can have negative and destructive effects on the broader society if not carefully deployed and monitored in an ethical manner. Consider the use and abuse of social media such as the internet or mobile phones for illegal and unethical activities such as luring children by pedophiles or the use of internet for financial scams and other illegal activities including terrorism. All of these untoward activities are probably deployed and used by individuals who may not recognize that such activities as unethical and immoral. All of these rapid changes in modern society including globalization, increased cross-cultural contact, climate change and environmental deterioration, the development and deployment of new technologies as well as the decline of traditional ethics and values, have magnified individual abilities to do good or bad, leading to new challenges in global bioethics.

## What needs to be done?

In light of the new challenges facing global bioethics in the 21^st ^century and beyond; the question has arisen about what needs to be done to expand the bioethics agenda especially in Africa and other developing countries. It has been suggested that the first thing to do is to develop competence in ethics, and to use this knowledge to deal with ethical issues that are facing humanity [9]. It has also been argued that a major mistake made by non-ethicists is to assume that the rightness or wrongness of a situation can be equated to the strength of our personal feelings. However, this non systematic approach is fraught with dangers, especially in the realm of new technologies where the individual developing the new technology in the laboratory is more concerned about the potential profits to be derived from the technology rather than possibility of its misuse and negative impact on society. Therefore training on ethics and systematic reflection and analysis of the social impact of rapid advances in science and their technological applications, has become a must, if these developments are to be applied to the benefit and not the detriment of society. For the developing countries in Africa, it is particularly important to build up competence in ethics for several reasons. Developing countries are exploited in so many ways through unfair trade agreements, supply of sub-standard products, bad treatment of workers [31], dumping of toxic wastes, biopiracy and takeover of natural resources, land, water, minerals, etc. Introduction of non-traditional crops or foods that displace local produce, e.g. GMO, corruption, bad governments, climate change, and depletion of environmental resources, all leading to poverty and changes in lifestyle [8,9,16-23].

## Teaching and learning ethics in Africa

It has been argued that the main challenges of teaching and learning ethics in Africa are related to the teaching content, when to teach, who will teach and the teaching methods [9,41,46]. It has been suggested that in order to accomplish the goals of teaching ethics, a student should become familiar with the structure of normative argumentation and the distinctions and knowledge required to arrive at sound ethical decisions [9]. This would involve the teaching of basic ethical norms or normative ethics to all students such as truth-telling, autonomy, consent, justice and human rights. An example of such approach is the UNESCO curriculum on Bioethics [47]. This should be followed by imparting ethical theories such as utilitarianism, virtue ethics and other aspects of deontological ethics. Further, students should be instructed in the ethical issues in various sciences such as environmental ethics, engineering ethics, medical and business ethics depending on the area of study. Other areas would involve research ethics and the history of ethics of science and medicine. We argue that another important area for students of ethics would be to impart information on poverty and its impact on developing nations to the students. Information on the goals and objectives of the millennium development goals (MDGs) [48], the goals and objectives of the newly proposed United Nations sustainable development, such as the goal of eradicating extreme poverty, as well as the demands for more justice, better accountability from elected public officials and an end to violence against women and other marginalized population groups [49]. Researchers and students can do much to help nations achieve the UN goals by focusing some of their projects and efforts on finding solutions. Creating of awareness of the needs of populations in the poorest nations may lead to a better approach at providing meaningful help and support to developing countries including those in Africa [48]. In terms of teaching, ethics training should be facilitated with concrete and practical examples in real life to illustrate abstract theories. Analyses by students can then be commented on by a trained teacher in ethics. One of the best ways of improving ethics teaching in Africa may be through an online format, thereby making it possible to teach large groups of students over long distances e.g. through massive online open classes (MOOCs) [50]. Perhaps introduction of the MOOCs format could be the best way for African higher education institutions to introduce generalized ethics teaching to a large number of African scholars. In this way, students can also continue with their work or other activities of normal life. It has been recommended by COMEST that universities and other institutions of higher education should be encouraged to teach ethics at three levels: (a) elementary ethics courses for all students (b) more advanced courses that are part of the PhD/ Masters graduate courses in the various sciences (c) courses that lead to a Ph.D. in ethics, suitable for people who already have a Ph.D. or are qualified in some other field e.g. medical specialists [9]. Finally, in terms of who should teach ethics, it has been suggested by COMEST that ethics courses be taught by teachers who have demonstrated research competence in ethics. While "ethics is of concern to all and we all have our views on ethical issues and like to express them, however, this does not qualify one to teach ethics" [9]. Teaching ethics is not the imparting of personal ethical views, but the enabling of others to take their independent stand on ethical issues. Many countries, especially developing countries do not have enough people with such qualifications. Therefore there is an urgent need in developing countries including those in Africa to build capacity in teaching ethics and to train more qualified teachers, especially those who would like to obtain additional qualifications in ethics" [9].

## Concluding comments

Africa is facing many ethical challenges in the 21^st ^century ranging from leadership, to organizational and professional ethics. One manifestation of this is corruption and brain drain, leading to loss of the much required human and physical resources for provision of desired services by state and private institutions. These problems have greatly affected the social responsibility and ability of many African states to provide adequate and essential services such as health care. Therefore many African states are yet to realize the full social responsibility and health as required by article 14 of the Universal Declaration on Bioethics and Human Rights [51]. The buck stops at the leadership in various sectors whether political or professional. It is considered unethical for the leadership to engage in practices that create conditions in which citizens are deprived of their right to basic services or force public service employees such as medical staff to strike so that their demands can be addressed. Today many African countries are off-track of the Abuja declaration requiring states to allocate at least 15% of their national budget to health sector [52]. It is true that there are many competing demands but one would expect better performance than at present levels. Africa is also faced with a high frequency of malpractice in clinical care in spite of current, albeit rudimentary regulatory frameworks. We have not done well in adherence to ethical standards and human rights. There is still low level of awareness among most of the population on individual rights to shape their destiny by making the right individual choices for their own well-being especially in the area of healthcare. Awareness paves way for capacity to make rational decisions. This may be occasioned by the fact that healthcare in Africa has remained in transition from traditional medicine to western medicine. Up until now it is estimated that majority of the rural population in Africa (up to 80%), still rely on traditional medicine [53]. Traditional medicine cannot be ignored since it complements the currently available conventional medicine, however one of the biggest challenges in Africa is poor quality, adulterated herbal and pharmaceutical products, caused by biopiracy, counterfeiting, and the quest for profit. To safeguard the users, we need adequate knowledge on safety, effectiveness and the quality of medicinal products and procedures. How do we deal with quacks in African traditional medicine? The debate in consenting to the unknown with respect to African herbal medicine is not likely to end soon. With increasing capacity to conduct research on herbal medicine and hence scientific proof of its potency we are likely to see an increase in harvesting of medicinal trees. This coupled with demand for such products in the west and east is a future threat to African biodiversity due to potential biopiracy. Africa is a fertile ground for pre and post clinical studies in biomedical research. The situation is complicated by the poverty in the region. Poor people are ready to trade their autonomy for daily bread when presented with an opportunity to participate in clinical trials and other forms of biomedical research. As a result, there is need to continue addressing the ethical issues of research related to healthcare in this region [45,54]. Another complex situation that faces Africa today is the global inequality in research capacity leading to skewed movement of life sciences and related specimens to the developed countries [55]. Whereas a significant development has been realized through initiation of national bioethics committees and institutional review boards among other agencies, issues of material transfer agreements are still poorly negotiated.

The rising number of regulatory agencies could have a double edge effect on the advancement of biomedical and biotechnology research in Africa. On a positive note, vulnerable groups and institutions may be protected from undue exploitation. On the negative side, excessive restriction can deny African institutions the much needed research capability to deal with emerging challenges facing health care providers in the context of limited resources. Until such time that the gross expenditure on research increases, the inequalities in research capacity will persist and more specimens will continue to be transported to developed world laboratories. There is unending debate on whether Africa should expressly accept GMO foods or products to deal with its perpetual food insecurity. There is no simplistic answer to ethical challenges facing the African continent. Bioethics is still at infancy in both in training and practice. There is also high vulnerability in the region, which is a consumer of scientific and technological advancement from the western world and more recently the east, in particular, China. Ethics has a crucial role in the development of science and technology in Africa. At the African Union in Addis Ababa in 2007, the former director general of UNESCO, Koïchiro Matsuura reiterated that the quest for sustainable development can never be achieved without ethical considerations [56]. Culturally, Africa had its own ways of conducting its affairs with utmost respect to humanity. These have been eroded by the so called modernization, erosion of the social fabrics of African societies by corruption and greed. All is not lost though, Africa still has the opportunity to rekindle its own ethics and catch up with the best ethical practices globally. In the final analysis, conferences such as the recently concluded *3^rd ^EHRML*- *African Health Congress *[57] hosted by Informa Health Sciences Exhibitions, should be encouraged because it enables African researchers and scientists to network with each other, discuss ethical and other healthcare issues affecting African communities and perhaps arrive at some African solutions to African problems. It is at similar conferences in other regions of the world that young researchers learn to debate and develop the skills in argumentation and to contest ideas based on the merits or demerits of the issues at stake [8]. These are the essential skills needed by African youth and researchers, if Africa is to make progress today and tomorrow. The papers in this supplement are part of that dialogue and debate and we hope that conferences such as the EHRML will continue in the future.

**Note: **The ideas and opinions expressed in this publication are those of the authors and do not necessarily represent the views of their respective or affiliated institutions.

*SCC- Durban, South Africa, August 22, 2013*.

## List of abbreviations used

COMEST: Commission on the ethics of scientific knowledge and Technology; EHRML: Ethics Human Rights and Medical Law; FCC: Federal Communications Commission; GMO: genetically modified organisms; MDGs: millennium development goals; MOOCs: Massive online open courses; RECS: Research Ethics committees, UN: United Nations, UNESCO: United Nations Educational Scientific and Cultural Organization; US: United States, USA: United States if America; ANFASA: Association of Non-Fiction Authors of South Africa.

## Competing interests

The authors declare that they have no competing interests.

## Authors' contributions

SCC conceptualized the idea and wrote the initial and final drafts of the manuscript. JK wrote the section on concluding comments and critically revised for important intellectual content. TM assisted in editing and critically revising the manuscript for intellectual content.

## Declarations

Publication of this supplement has been funded by the College of Health Sciences and the Research Office at the University of Kwazulu-Natal.

This article has been published as part of *BMC Medical Ethics *Volume 14 Supplement 1, 2013: Selected papers from the 3rd Ethics, Human Rights and Medical Law Conference (3rd EHMRL). The full contents of the supplement are available online at http://www.biomedcentral.com/bmcmedethics/supplements/14/S1.
